# *Clonorchis sinensis* extracellular vesicles associated with Csi-let-7a-5p activate pro-inflammatory macrophages to induce biliary injury

**DOI:** 10.1371/journal.pntd.0013080

**Published:** 2025-05-13

**Authors:** Beibei Zhang, Xing Li, Qian-Yang Zhou, Chen Zhang, Zheng-Rui Bian, Xin-Xin Ren, Qian Yu, Hui Hua, Zhihua Jiang, Bo Zhang, Xiang-Yang Li, Mu-Xin Chen, Kui-Yang Zheng, Chao Yan

**Affiliations:** 1 Jiangsu Key Laboratory of Immunity and Metabolism, Jiangsu International Key Laboratory of Immunity and Metabolism, National Demonstration Center for Experimental Basic Medical Science Education, Department of Pathogenic Biology and Immunology, School of Basic Medical Science, Xuzhou Medical University, Xuzhou, People’s Republic of China; 2 Department of Dermatology, The Second Affiliated Hospital of Nanjing Medical University, Nanjing, China; 3 Institute of Parasitic Disease Control and Prevention, Guangxi Key Laboratory for the Prevention and Control of Viral Hepatitis, Guangxi Zhuang Autonomous Region Center for Disease Control and Prevention, Nanning, China; 4 National Key Laboratory of Intelligent Tracking and Forecasting for Infectious Diseases, National Institute of Parasitic Diseases, Chinese Center for Disease Control and Prevention (Chinese Center for Tropical Diseases Research), NHC Key Laboratory of Parasite and Vector Biology, Shanghai, People’s Republic of China; 5 Hainan Tropical Diseases Research Center (Hainan Sub-Center, Chinese Center for Tropical Diseases Research), Haikou, People’s Republic of China; Zhejiang Wanli University, CHINA

## Abstract

During *Clonorchis sinensis* (*C. sinensis*) infection, pro-inflammatory macrophages (M1 macrophages) are highly activated, yet their role in the disease remains poorly understood. Previous studies have demonstrated that extracellular vesicles from *C. sinensis* (CsEVs) can activate these macrophages, and inhibiting a specific miRNA (Csi-let-7a-5p) in CsEVs (InCsEVs) can reduce this activation. In the present study, liver macrophages in mice were removed using clodronate liposomes (Clodlip). Subsequently, different types of bone marrow-derived macrophages (BMDMs) were adoptively transferred into the mice lacking liver macrophages: untreated (PBS-BMDM), treated with CsEVs (CsEVs-BMDM), treated with a control (ScrCsEVs-BMDM), or treated with InCsEVs (InCsEVs-BMDM). Biliary damages were then evaluated. The results indicated that the transferred macrophages successfully repopulated the mice. CsEVs-BMDM led to significant inflammation and bile duct damage, accompanied by higher levels of inflammatory cytokines (TNF-α and IL-1β). However, when macrophages were treated with InCsEVs, the damage and inflammation were alleviated, and the levels of TNF-α and IL-1β decreased. These findings suggest that pro-inflammatory macrophages activated by CsEVs, especially through Csi-let-7a-5p, play a crucial role in biliary damage during *C. sinensis* infection. Although other immune cells may also be involved, this study emphasizes the significance of pro-inflammatory macrophages in clonorchiasis.

## Author summary

Clonorchiasis, caused by the liver fluke *Clonorchis sinensis*, triggers chronic inflammation and bile duct damage. Pro-inflammatory macrophages are implicated, but their exact role was unclear. In this study, we removed liver macrophages in mice using clodronate liposomes. Donor macrophages (BMDMs) were activated to a pro-inflammatory state using *C. sinensis*-derived extracellular vesicle (CsEVs) or treated to block a key miRNA (Csi-let-7a-5p) to reduce inflammation (InCsEVs-BMDM). These macrophages were transferred into mice lacking their macrophages. We found that CsEVs-activated macrophages caused severe bile duct damage and inflammation. Blocking Csi-let-7a-5p (InCsEVs-BMDM) reduced inflammation and damage. The parasite’s particles (CsEVs) drive bile duct injury by overactivating pro-inflammatory macrophages.

## Introduction

Clonorchiasis caused by *Clonorchis sinensis* is one of the most severe neglected tropical diseases [1]. This fluke dwells in the biliary duct of various mammals such as humans and cats, which can lead to liver damage, cholangitis, bile duct hyperplasia, gallstones, periductal hepatic fibrosis, and even cholangiocarcinoma [2]. Although this parasite importantly affects human health, the pathogenesis caused by this worm is largely unknown.

As innate immune cells, macrophages have multiple functions in fighting against infection, immunopathological injury, tissue repair, and regeneration [3]. Although macrophages are very heterogeneous and their functions are tissue-specific and finely regulated by the local micro-environment, they have been generally classified as pro-inflammatory macrophages (M1) and anti-inflammatory macrophages (M2) for a long time, which represent two basic functions of macrophages [3]. During *C. sinensis* infection, macrophages are also activated and extensively participate in fighting against worms’ invasion, and immunopathological damage due to the pro-inflammatory cytokines/ mediators produced by M1 [4]. Of these activated macrophages, pro-inflammatory macrophages can be activated by excretory-secretory products (ESPs) of *C. sinensis* (CsESPs), extracellular vesicles of *C. sinensis* (CsEVs), and some proteins such as CsMF6p/HDM [5–7]. Interestingly, while *F. hepatica* infection suppressed pro-inflammatory M1 macrophages, *C. sinensis* significantly activated pro-inflammatory macrophages in both acute and chronic infections [4]. This suggests that pro-inflammatory M1 macrophages play pleiotropic roles during *C. sinensis* infection [4,8,9]. These different patterns of activation of pro-inflammatory M1 macrophages may also account for the different pathology caused by these liver flukes.

Extracellular vesicles (EVs) represent a phospholipid membrane-enclosed vesicle secreted by living cells into extracellular space. EVs are now emerging as important vehicles of cell-cell communication as they can deliver molecular signals such as RNAs, proteins, and lipids sourced from parent cells to the target cells over a long distance. Helminthes, the multicellular organisms, also secret EVs contained in their released ESPs [10]. Studies have demonstrated that EVs from helminth are also important mediators in the interaction between helminth and host, which induce a broad spectrum of physio-pathological functions, including facilitation of worms’ survival, induction and modulation of immune responses, and promoting host tissue damage or repairment [11–13]. For example, our previous study demonstrated that extracellular vesicles from *C. sinensis* (CsEVs) can potently induce a pro-inflammatory phenotype of macrophage and suppress the activation of anti-inflammatory macrophage; furthermore, a miRNA Csi-let-7a-5p packaged by CsEVs was identified to be critical in inducing pro-inflammatory activation of macrophage caused by CsEVs, whereas the inhibition of Csi-let-7a-5p in CsEVs attenuated the activation of pro-inflammatory macrophage, thus ameliorating the biliary damage caused by CsEVs [5]. However, the roles of pro-inflammatory macrophages activated by CsEVs remain obscure. In the present study, we depleted the macrophage in the recipient mice using clodronate liposome and then adoptively transferred CsEVs-activated pro-inflammatory macrophages. We aimed to investigate whether these pro-inflammatory macrophages are directly involved in biliary damage. We found that these adoptively transferred pro-inflammatory macrophages can still maintain the pro-inflammatory property in the liver of recipient mice and trigger biliary damage, as well as bile duct hyperplasia. Our study highlights the importance of the pro-inflammatory macrophages as an important mediator that contributes to pathogenesis caused by *C. sinensis* infection.

## Results

### CD45.1^+^ macrophages were successfully transferred and reconstructed in the liver of CD45.2^+^ mice

**Fig 1:**
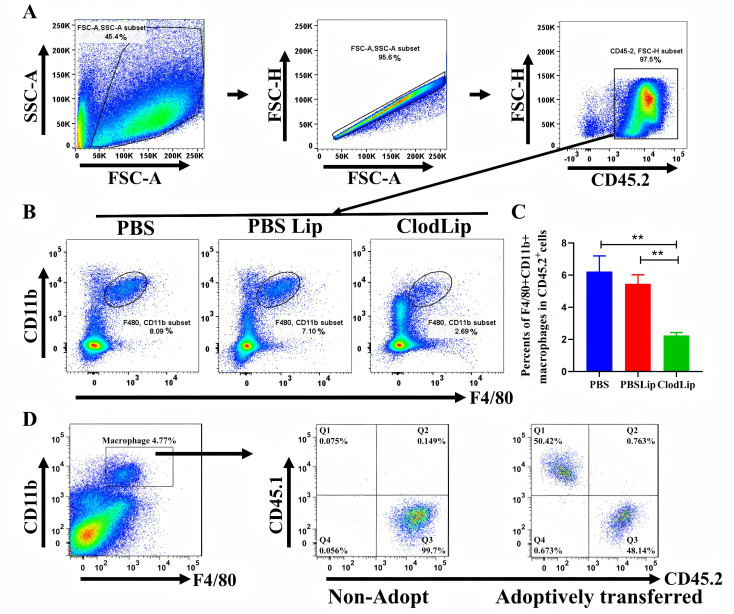
The depletion of CD45.2^+^ macrophages and adoptive transfer of CD45.1^+^ macrophages to CD45.2^+^ recipient mice. **A&B**: The gating strategy and percentage of macrophages by flow cytometry and the representative scatter plot of macrophages in the liver of mice that were administrated by PBS, PBS liposomes, or clodronate liposomes; **C**: the percent of CD45.2^+^ macrophages in the livers of mice; **D**: The percent of CD45.1^+^ macrophages (adoptively transferred) and CD45.2^+^ macrophages (residential macrophages from recipient mice). n = 4 ~5, One-way ANOVA with LSD test was used. Compared with the corresponding group, **P < 0.01.

Our previous study demonstrated that CsEVs packaging Csi-let-7a-5p triggered pro-inflammatory activation of macrophages [5]. However, the roles of macrophages activated by CsEVs in biliary injury were still obscure. To address this, we employed clodronate liposome (Clodlip) to deplete macrophages in CD45.2^+^ mice, and BMDMs (S1 Fig) from CD45.1^+^ donor mice were adoptively transferred to substitute and reconstruct macrophages in the CD45.2^+^ mice *via* tail vein. Flow cytometry data showed that the percentage and numbers of macrophages (F4/80^+^CD11b^+^) in the livers of Clodlip-treated mice were significantly decreased, compared with PBS containing liposome treated mice (PBSLip group) or PBS-only treated mice (PBS group, Fig 1A–1C for the percentage of macrophages, *P* < 0.01; S2 Fig. A and B for numbers of macrophages), suggesting that the macrophages in the recipient mice were effectively depleted. Next, we evaluated whether xenografted CD45.1^+^ labeled macrophages can be reconstructed in the liver of recipient mice or not. As shown in Fig 1D, it showed that among all the macrophages, about 50% of macrophages were CD45.1^+^ macrophages, while the other half were original macrophages from the recipient mice (Fig 1D). All these data indicate that CD45.1^+^labeled macrophages were successfully transferred and reconstructed in the liver of CD45.2^+^ mice.

### Adoptive transfer of macrophages activated by CsEVs induced hepatic bile duct injury in mice

**Fig 2:**
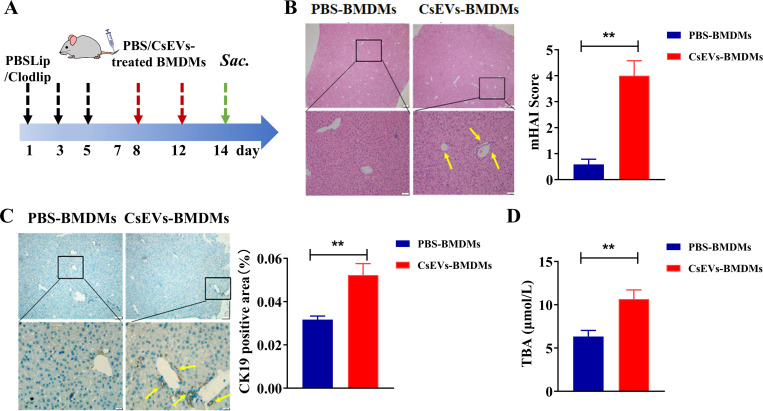
Pathological damage of liver and bile ducts in mice adoptively transferred with PBS-BMDMs or CsEVs-BMDMs. **A**: Schematic diagram of administration of mice with macrophages; **B**: H&E staining (×40, × 200) shows pathological scores of liver pathological sections of mice in each group; **C**: Positive distribution of CK19 (×40, × 200) and CK19 in liver sections of mice in each group. Semi-quantitative analysis of positive distribution; **D**: serum TBA in mice. n = 6, compared with the corresponding groups, a paired student’s *t*-test was used for comparison. Compared with the indicated group, **P < 0.01.

To evaluate the pathogenesis of pro-inflammatory macrophages activated by CsEVs in biliary injuries, we adoptively transferred these macrophages to the recipient mice that have been depleted by Clodlip or PBSLip according to the strategy shown in Fig 2A. H&E staining showed that there were no obvious lesions around the central vein and portal area in the PBS-BMDMs group. However, compared with the mice transferred with PBS-treated BMDMs, a large number of inflammatory cells were observed around the portal area of the mice in the CsEVs-BMDMs transferred mice (Fig 2B, *P* < 0.01). The expression of CK19, a marker indicating biliary hyperplasia in mice, was detected by immunohistochemistry. The results showed that compared with the control mice, the positive distribution areas of CK19 in the livers of the mice in the CsEVs-BMDMs were significantly increased (Fig 2C, *P* < 0.01). We also detected sera indicators of cholestasis such as total bile acid (TBA), we found that the mice adoptively transferred with the CsEVs-BMDMs group had a higher level of TBAs than those mice transferred with PBS-treated BMDMs (Fig 2D, *P* < 0.01). These data suggest that the transferred pro-inflammatory BMDMs activated by CsEVs can induce biliary damage.

### The transferred CsEVs-activated BMDMs remained M1 activation in the recipient mice

**Fig 3:**
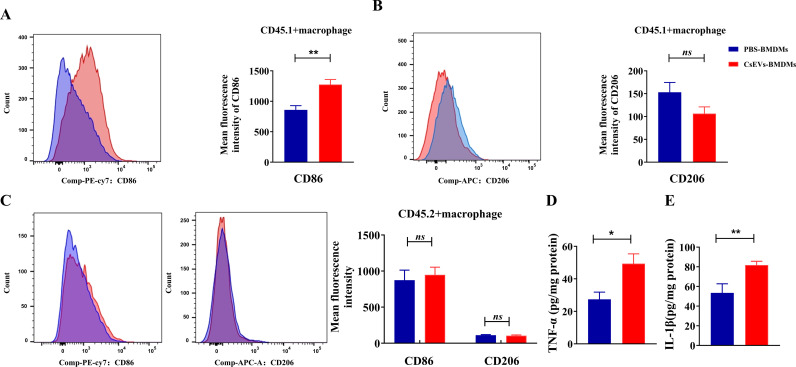
The adoptively transferred macrophages maintained M1 activation in the liver of recipient mice. The CD45.2^+^ mice were administrated with PBS-BMDMs or CsEVs-BMDMs twice as shown in Fig 2, the mice were sacrificed, and F4/80^+^CD11b^+^were used to define the macrophages within the gates of CD45.1^+^ or CD45.2^ +^ cells. **A**: the levels of CD86 in the CD45.1^+^ macrophages; **B**: the levels of CD206 in the CD45.1^+^ macrophages; **C**: the levels of CD86 and CD206 in the CD45.2^+^ macrophages; **D**: The concentrations of TNF-α in the liver of recipient mice; **E**: The concentrations of IL-1β in the liver of recipient mice. n = 6, a paired student’s *t*-test was used for comparison. Compared with the indicated groups, *P < 0.05, **P < 0.01, ns means no statistical difference.

To evaluate the activation of liver macrophages that were adoptively transferred, we detected the levels of CD86 (M1) or CD206 (M2) of macrophages (CD11b^+^F4/80^+^) in the livers of the recipient mice. The flow cytometry data showed that compared with the PBS-BMDMs group, the levels of CD86 in the CD45.1^+^ macrophages from the liver with the CsEVs-BMDMs adoptively transferred was significantly increased (Fig 3A, *P* < 0.01). However, we found that there was no statistical significance of CD206 between PBS-BMDMs and CsEVs-BMDMs groups, although there was a slight decrease in the CsEVs-BMDMs in the recipient mice (Fig 3B, *P* > 0.05). Furthermore, we also found that there was no significant change in the expression of CD86 or CD206 in CD45.2^+^ macrophage mice between PBS-BMDMs and CsEVs-BMDMs groups (Fig 3C, *P* > 0.05). These data suggested that macrophages originating from the recipient mice were not activated due to the adoptive transfer, and the biliary damage was not likely caused by CD45.2^+^ macrophages.

In addition, we also detected the expression of pro-inflammatory cytokines TNF-α and IL-1β in mouse liver tissue using ELISA. We found that, compared with the PBS-BMDMs transferred mice, the levels of TNF-α and IL-1β in the liver tissues of CsEVs-BMDMs transferred mice were significantly increased (Fig 3D for TNF-α, *P* < 0.05; Fig 3E for IL-1β, *P* < 0.01). These data indicated that the adoptively transferred macrophages activated by CsEVs remained pro-inflammatory and may be involved in the biliary damage.

### Suppressed pro-inflammatory activation of BMDMs induced by InCsEVs attenuates the hepatic bile duct injury in mice

**Fig 4:**
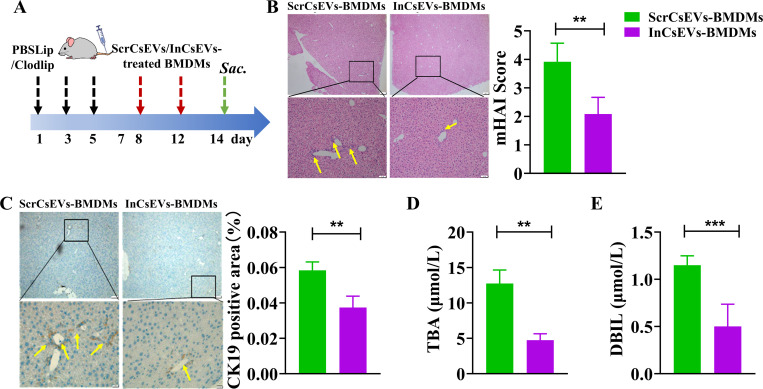
The biliary injuries and cholestasis are alleviated by the decreased pro-inflammatory activation of macrophages treated with InCsEVs. BMDMs were stimulated with Scramble-CsEVs or CsEVs with inhibition of Csi-let-7a-5p (InCsEVs) for 24 h, BMDMs were collected for adoptively transferred as shown in **A**, the mice adoptively transferred by ScrCsEVs-BMDMs or InCsEVs-BMDMs were sacrificed on the indicated day; H&E staining was analyzed in **B**; **C**: the expression of CK19 was evaluated by IHC staining; **D**: the concentration of total bile acids in the sera of the recipient mice; **E**: the concentration of direct bilirubin (DBIL) was detected in the sera of the recipient mice that were adoptively transferred by ScrCsEVs-BMDMs or InCsEVs-BMDMs. n =  6, a paired student’s *t*-test was used for comparison. Compared with the indicated groups, **P < 0.01, ***P < 0.001.

Our previous study identified a miRNA, Csi-let-7a-5p, as a cargo of CsEVs, which contributes to the pro-inflammatory activation of macrophages induced by CsEVs as we found that the inhibition of Csi-let-7a-5p in CsEVs by RNAi (InCsEVs) can abrogate the pro-inflammatory activation [5]. Based on this finding, we adoptively transferred the macrophages with restrained pro-inflammatory activation induced by InCsEVs to the recipient mice and evaluated the biliary injuries and local inflammation (Fig 4A). H&E staining showed that ScrCsEVs-treated BMDMs triggered immune cell infiltration and a small amount of punctate necrosis in the liver parenchyma of the recipient mice, but in the InCsEVs-BMDMs transferred mice, the infiltrated immune cells and the areas of punctate necrosis were significantly decreased as indicated by mHAI score (Fig 4B, *P* < 0.01). We also evaluated the biliary hyperplasia using the marker of CK19 by IHC staining. The data showed that CK19-positive areas in the livers of InCsEVs-BMDMs transferred mice were significantly reduced, compared with the ScrCsEVs-BMDMs transferred mice (Fig 4C, *P* < 0.01). Furthermore, it was found that the concentrations of TBA and DBIL in the sera of InCsEVs-BMDMs transferred mice were significantly lower than those in ScrCsEVs-BMDMs transferred mice (Fig 4D for TBA, *P* < 0.01; Fig 4E for DBIL, *P* < 0.001). Summarily, adoptively transferring the decreased pro-inflammatory activation of macrophages alleviated biliary injuries and cholestasis.

### The suppression of pro-inflammatory activation of macrophages induced by InCsEVs in the livers of recipient mice

**Fig 5:**
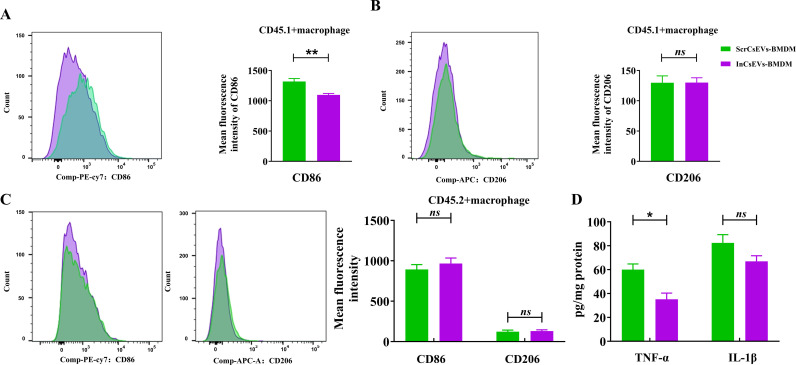
The decreased inflammation in the liver of the CD45.2^+^ recipient mice that were adoptively transferred with InCsEVs-BMDMs. The recipient mice were administrated by ScrCsEVs-BMDMs or InCsEVs -BMDMs as shown in Fig 4B. **A**: the expression of CD86 in the CD45.1^+^ macrophages (F4/80^+^CD11b^+^) was detected by flow cytometry; **B**: the expression of CD206 in the CD45.1^+^ macrophages in the liver of recipient mice; **C**: the expression of CD86 and CD206 in the CD45.2^+^ macrophages in the liver of recipient mice; **D**: the concentrations of TNF-α and IL-1β in the livers of recipient mice. n =  6, a paired student’s *t*-test was used for comparison. Compared with the indicated groups, *P < 0.05, **P < 0.01, *ns* means no statistical difference.

To evaluate the activation of macrophages in the recipient mice after adoptive transfer with ScrCsEVs or InCsEVs-macrophages, we isolated the hepatic macrophages and employed the flow cytometry to detect the levels of CD86 or CD206 in either CD45.1^+^ or CD45.2^+^ macrophages. The data showed that there was a significant decrease in the levels of CD86 between the transferred ScrCsEVs-macrophages and InCsEVs -macrophages as indicated by CD45.1^+^ (Fig 5A, *P* < 0.01). However, there was no significant difference in the expression of CD206 in transferred macrophages between these two groups (Fig 5B, *P* > 0.05). Furthermore, we didn’t find a difference in the expression of CD86 nor CD206 in the CD45.2^+^ macrophages, suggesting that adoptive transferred with ScrCsEVs or InCsEVs -macrophages could not induce the activation of macrophages sourced from recipient mice (Fig 5C, *P* > 0.05). Compared with the ScrCsEVs-macrophages, the concentrations of TNF-α in the livers of InCsEVs-macrophages transferred mice were significantly decreased (Fig 5D for TNF-α, *P* < 0.05). In addition, we also found that there was a declining trend of IL-1β in the InCsEVs -macrophages transferred mice, compared with those in the livers of ScrCsEVs transferred mice although a statistical significance was not observed (Fig 5D for IL-1β, *P* > 0.05). Collectively, these data demonstrate that the decreased pro-inflammatory activation of adoptively transferred macrophages due to treatment with InCsEVs but not native macrophages of the recipient mice is critical to the amelioration of biliary injuries and cholestasis.

## Discussion

**Fig 6:**
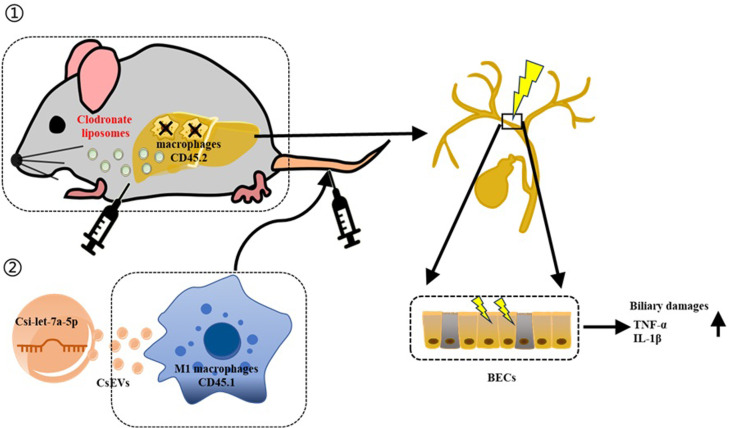
Pro-inflammatory macrophages activated by CsEVs delivering Csi-let-7a-5p induce biliary damages.

The hepatic macrophages represent one of the main innate immune cells that are critical to the development and resolution of liver diseases [14–16]. Generally, there are two basic subpopulations of macrophages due to their activations and functions: pro-inflammatory macrophages and anti-inflammatory macrophages. Regarding the liver fluke infection, a recent review summarized and compared the different responsive patterns of macrophages to *O. viverrini* and *C. sinensis* vs *F. hepatica* [4,8]. It suggests that pro-inflammatory M1-like macrophages caused by *C. sinensis* or *O. viverrini* might drive the mutagenesis to support tumor initiation [4]. However, EVs from another liver fluke-*Fasciola hepatica* reduced the migratory capacity of monocytes and induced a mixed M1/M2 response, exerting anti-inflammatory properties in the inflammatory conditions [12]. Indeed, macrophages are dynamically changed during *C. sinensis* infection. M1 macrophages increase at the early stage and chronic stages, while M2 macrophages (CD206^+^ Macrophages) increase at the chronic stage of *C. sinensis* infection [8]. However, the role that macrophages play in biliary injury remains incompletely defined. Herein, in the present study, the hepatic macrophages in the CD45.2^+^ mice were depleted by liposome chlorophosphite, and reconstructed by adoptive transferring of pro-inflammatory BMDMs that were activated by CsEVs containing Csi-let-7a-5p or depressed pro-inflammatory BMDMs by InCsEVs. It was found that the pro-inflammatory macrophages in the liver of recipient mice triggered the biliary damage and bile duct hyperplasia. This highlights that pro-inflammatory macrophages activated by CsEVs carrying Csi-let-7a-5p contribute to the pathogenesis of *C. sinensis* infection (Fig 6).

It has been demonstrated that extracellular vesicles (EVs) secreted by helminths can mediate the distant communication between host cells and worms by delivering bioactive molecules such as miRNAs, proteins, and lipids [17]. It has also been shown that extracellular vesicles (EVs) have various pathophysiology functions during infection, including inhibiting pathogen survival, regulating the host immune response, and promoting host tissue repair/regeneration [11,18]. In our previous study, we had well-established a method to isolate CsEVs from the cultures of worms and demonstrated that CsEVs can potently induce the activation of macrophages which leads to biliary injuries [5]. Other studies also found that CsEVs can activate the cholangiocytes to produce inflammatory cytokines such as TNF-α and IL-6 via TLR9 or TLR3 mediated p38/ERK signaling pathway [19,20]. These data highlight the importance of CsEVs as the mediators in the regulation of immune responses during worm infection.

miRNAs can be involved in the regulation of macrophage activation. For example, it has been found that cholesterol-induced lysosomal dysfunction increases exosome release from hepatocytes, resulting in M1 polarization and macrophage-induced inflammation in a miR-122-5p-dependent manner [21]. Our previous study demonstrated that Csi-let-7a-5p enriched in CsEVs could prefer the activation of pro-inflammatory macrophages and facilitate biliary injuries by targeting the *Clec7a*- and *Socs1*-modulated NF-κB signaling pathways [5]. Consistent with previous data, we found that CsEVs can induce the pro-inflammatory activation of macrophages as indicated by the high levels of IL-6 and TNF-α, however, when the expression of Csi-let-7a-5p was inhibited, the levels of these cytokines secreted by BMDMs were decreased, accordingly. All these indicate that Csi-let-7a-5p is one of the main contents in the CsEVs to regulate the activation of the pro-inflammatory macrophages although the roles of let-7a-5p in different kinds of diseases may be context-dependent [22,23].

To decipher the role of macrophages in biliary injury, it could be necessary to establish a model of hepatic macrophage depletion and reestablishment. The use of chlorophosphite can reduce the macrophages in the injured liver in a BDL model, suggesting that injection of chlorophosphite is an effective method to diminish the macrophages in the liver [24]. Chlorophosphite is a small hydrophilic bisphosphonate molecule, once encapsulated in liposomes, which can be quickly recognized and swallowed by hepatic macrophages, permitting an efficient threshold of clodronate concentration to trigger apoptosis of the targeted cells [25,26]. The clearance of macrophages was up to 50% in our present study as evaluated by flow cytometry, which achieved the effects of liposome on macrophages’ deletion [27]. And the transferred macrophages could maintain their function for a long time (at least 14 days) [28]. Furthermore, the residual macrophages did not change significantly after adoptively transferred macrophages, indicating that the adoptively transferred macrophages but not resident macrophages contributed to the biliary injuries. However, since clodronate liposomes can eliminate all types of macrophages in the body, it cannot be ruled out that the macrophages in other tissues, such as the intestine, lung, etc. may also be depleted in our present study [29]. In our present study, we focused on the roles of macrophages in the biliary damages induced by CsEVs, however, other kinds of infiltered immune cells such as neutrophils may participate in the pathogenesis caused by CsEVs. In summary, the present study demonstrates that pro-inflammatory macrophages activated by CsEVs induce biliary injuries, which highlights the importance of pro-inflammatory macrophages in the pathogenesis of clonorchiasis.

## Materials and methods

### Ethics approval and consent to participate

Animal care and all experiments performed in this study were strictly conformed to the guidelines of the National Laboratory Animal Center. The main procedures and protocol were reviewed and approved by the Animal Care and Use Committee of Xuzhou Medical University License (201701w007).

### Animals

Specific pathogen-free (Specific pathogen Free, SPF) grade, female C57BL/ 6J mice (CD45.2^+^ mice) with the age of 6~8 weeks were purchased from Charles River Experimental Animal Technology Co., Ltd. [SCXK (Zhejiang) 2019–0001]. Female, C57BL/6J SJR CD45.1^+^ mice aged 6~8 weeks were generously gifted from Professor Hui Wang from Xuzhou Medical University. All mice were raised in SPF facilities with free access to food and water. All the operations such as tail vein injection were performed in the animal barrier experiment room of the Experimental Animal Center of Xuzhou Medical University [SYXK (Su) 2016–0028]. 

### Worm culture and RNAi

The adult worms were obtained from *C. sinensis*-infected guinea pigs-a well-documented animal model for *C. sinensis* infection [30]. In brief, a guinea pig model of *C. sinensis* infection was set out, then the adult worms were collected after 8 weeks of infection, and the adult worms with high vitality were maintained in RPMI-1640 medium containing 1% (v/v) penicillin/streptomycin without phenol sulfonphthalein. To obtain the depressed Csi-let-7a-5p in CsEVs, RNA interference (RNAi) assays in cultured worms were performed according to the reference [5]. The specific double-stranded RNA of Csi-let-7a-5p and scramble were manufactured by GenePharma (GenePharma, Shanghai, China), and sequences are as follows: Csi-let-7a-5p inhibitor: 5‘-ACCACACAACGAACUACCUCC-‘3; scramble: 5’- CAGUACUUUUGUGUAGUACAA-‘3. The worms were cultured with RPMI-1640 medium containing siRNA (50 ng/μl) solution or its scrambled control (50 ng/μl) solution for 24 h, and then the supernatant was discharged and the live worms were washed 3 times using PBS. The live worms were further cultured in RPMI-1640 medium for 24 h to prepare ESPs produced by PBS, scramble, or siRNA-treated worm.

### Preparation of CsEVs, ScrEVs, and InEVs

CsEVs, ScrCsEVs, or InCsEVs were isolated from ESPs of *C. sinensis* treated by PBS, scramble, or siRNA of Csi-let-7a-5p as described elsewhere, respectively [5]. Briefly, ESPs from PBS, scramble, or siRNA-treated worms were collected and CsEVs, ScrCsEVs, or InEVs were prepared by differential centrifugation with 1 h 45 min at 120 000 g for twice. After centrifugation, the sediment was dissolved in 100 μl PBS. These different sourced CsEVs were aliquoted and stored at -80 °C for further use.

### Bone marrow-derived macrophage (BMDM) culture

The CD45.1^+^ BMDMs of C57BL/6J SJR mice were routinely isolated and cultured, then stimulated with PBS, CsEVs (32 μg/ml), Scrambled-CsEVs (32 μg/ml) and Inhibitor-CsEVs (32 μg/ml) for 24 h to obtain PBS-BMDMs, CsEVs-BMDMs, ScrCsEVs-BMDMs, and InCsEVs-BMDMs, respectively. The cells were collected to adaptively transfer to the receipt mice by the following procedures.

### Animal administration

C57BL/6J mice were randomly divided into 3 groups in our study as follows: PBS group (n = 5), PBS liposomes-treatment group (PBSLip, n = 4), and clodronate liposomes-treatment group (ClodLip, n = 28). On the 1st, 3rd, and 5th day, PBS, PBS liposomes, or clodronate liposomes were injected via the tail vein respectively with 100 μl per mouse. 4 mice selected randomly from the ClodLip group or PBSLip group were sacrificed and their livers were collected to detect the clearance of macrophages by flow cytometry. The remaining 24 mice in the ClodLip group were randomly divided into 4 groups, on the 8th and 12th day, they were administrated by different stimulated cells (PBS-BMDMs, CsEVs-BMDMs, ScrCsEVs-BMDMs or InCsEVs-BMDMs by tail vein injection, n = 6) with 1.5 × 10^6^ cells per mouse. On the 14th day, the mice were sacrificed, and the serum collected from the orbital sinus and liver tissue was collected for further study.

### Flow cytometry

The hepatic macrophages were detected as the previous report [31]. In brief, the liver was homogenated, and non-parenchyma cells were isolated with 40% and 70% Percoll (GE Healthcare 17-0891-01), followed by after lysis of red blood cells (BioLegend Cat. No. 420301). Flow cytometry was applied to assess the clearance of the liver macrophages (F4/80^+^CD11b^+^) by gating with CD45.2^+^ or CD45.1^+^. The polarization of macrophages was evaluated by the makers CD86 or CD206 of adoptively transferred macrophages. For flow cytometry detection, Fc receptors of macrophages were blocked using anti-mouse CD16/32 (TruStain Fc PLUS, BioLegend, USA). And the following antibodies were used to stain cell surface markers of isolated liver macrophages: PE anti-mouse CD45.1(Cat#:110707, BioLegend, USA); FITC anti-mouse CD45.2 (Cat#109805; BioLegend, USA), BV 510 anti-mouse CD11b (Cat#101263, BioLegend, USA); BV421 anti-mouse F4/80 (Cat#123131, BioLegend, USA); PE/Cy7 anti-mouse CD86 (Cat#105014, CST, USA); Alexa 647 anti-mouse CD206 (Cat#565250, BD, USA). For counting the number of macrophages, the CountBright beads (Cat#C36950, Invitrogen, USA) were added into the tubes according to the instructions. The samples were detected by flow cytometer (FACS Canto II, BD, USA), and analyzed by FlowJo software.

### Histology and immunohistochemistry

The liver tissue was fixed, dehydrated, and transparent, then embedded in paraffin, and serially sectioned at 4 μm for hematoxylin and eosin staining (HE) or immunohistochemistry (IHC). Briefly, for HE staining, after sealing the slides with neutral adhesive, the histological examination of stained histological sections was inspected under light microscopy (Olympus, Japan). The pathological scoring was assigned to each slide according to the reference with the criteria [32]. For IHC staining, deparaffinized sections were incubated with 3% H_2_O_2_ for 10 min at room temperature to quench the endogenous peroxidase activity. Sections were blocked with 5% goat serum for 1 h and then overnight at 4˚C with rabbit anti-mouse CK19 (diluted 1:400, Cat#ab193600, Abcam, US) or Ki67 (1:300, 12292S, CST, US) primary antibodies; after washed with PBS, the appropriate biotin-conjugated secondary antibody (1:1000 dilution) was added to incubate at room temperature for 1 h, after twice washing, DAB was added. The sections were observed under the microscope (×200 magnifications) and the fields were randomly selected from each section and CK19 positive areas were calculated by Image-Pro Plus 6.0 software (IPP, Media Cybernetics, Inc., USA).

### Serum biochemical assay

Serum biochemical measurements for total bilirubin (TBIL) and total bile acid (TBA) were assayed by the Department of Laboratory Medicine, Affiliated Hospital of Xuzhou Medical University in China.

### Enzyme-linked immunosorbent assay

The liver tissue was homogenated in RIPA buffer supplemented with protease and phosphatase inhibitors, the supernatants were collected and the protein levels of TNF-α and IL-1β were determined by ELISA, according to the manufacturer’s instructions.

### Statistical analysis

All statistics were performed with SPSS 16.0 software, all the data were presented as mean±s.e.m. The two-tailed student *t*-test was used for comparison between the two groups. One-way ANOVA following the Least Significant Difference (LSD) test was used for comparison of more than two groups. *P* < 0.05 was considered the difference to be statistically significant.

## Supporting information

S1 Fig BMDMs were successfully induced from bone marrow cells. **(A)** the observation of the morphology of BMDMs under the microscope; **(B)** flow cytometry analysis of F4/80^ +^ CD11b^+^macrophages gating with CD45.2^ +^ cells.
(DOCX)

S2 Fig The depletion of macrophages in the liver of mice using clodronate liposome. **(A)** the black arrows show the gating strategy of the macrophages. The red arrows show the signal of beads for counting cells; **(B)** the absolute numbers of macrophages in the liver after PBS liposomes (PBS Lip) or clodronate liposomes (ClodLip) treatment. n = 4~5 mice per group. Compared with the corresponding group, **P < 0.01, ns means no statistical differences.
(DOCX)
