# Supplementary material for: Clonorchis sinensis extracellular vesicles associated with Csi-let-7a-5p activate pro-inflammatory macrophages to induce biliary injury
Source: PLoS Negl Trop Dis. 2025 May 13;19(5):e0013080. doi: 10.1371/journal.pntd.0013080 (PMC12074333; doi:10.1371/journal.pntd.0013080)
Supplement: S1 Fig — (A) the observation of the morphology of BMDMs under the microscope; (B) flow cytometry analysis of F4/80 + CD11b+macrophages gating with CD45.2 + cells. (DOCX) [file pntd.0013080.s001.docx]

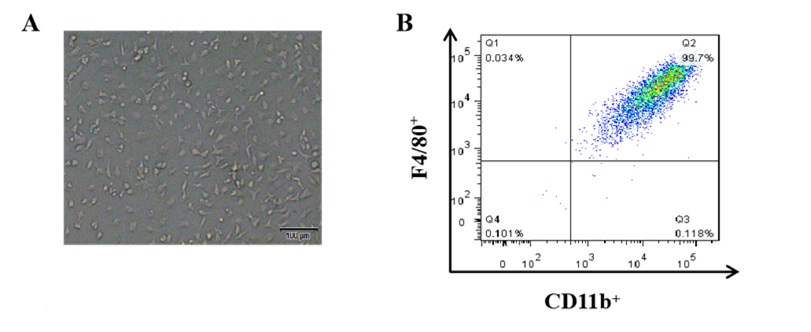


**S1 Fig. BMDMs were successfully induced from bone marrow cells.** **(A)** the observation of the morphology of BMDMs under the microscope. **(B)** flow cytometry analysis of F4/80+CD11b+macrophages gating with CD45.2+ cells.
