# Supplementary material for: Clonorchis sinensis extracellular vesicles associated with Csi-let-7a-5p activate pro-inflammatory macrophages to induce biliary injury
Source: PLoS Negl Trop Dis. 2025 May 13;19(5):e0013080. doi: 10.1371/journal.pntd.0013080 (PMC12074333; doi:10.1371/journal.pntd.0013080)
Supplement: S2 Fig — (A) the black arrows show the gating strategy of the macrophages. The red arrows show the signal of beads for counting cells; (B) the absolute numbers of macrophages in the liver after PBS liposomes (PBS Lip) or clodronate liposomes (ClodLip) treatment. n = 4~5 mice per group. Compared with the corresponding group, **P < 0.01, ns means no statistical differences. (DOCX) [file pntd.0013080.s002.docx]

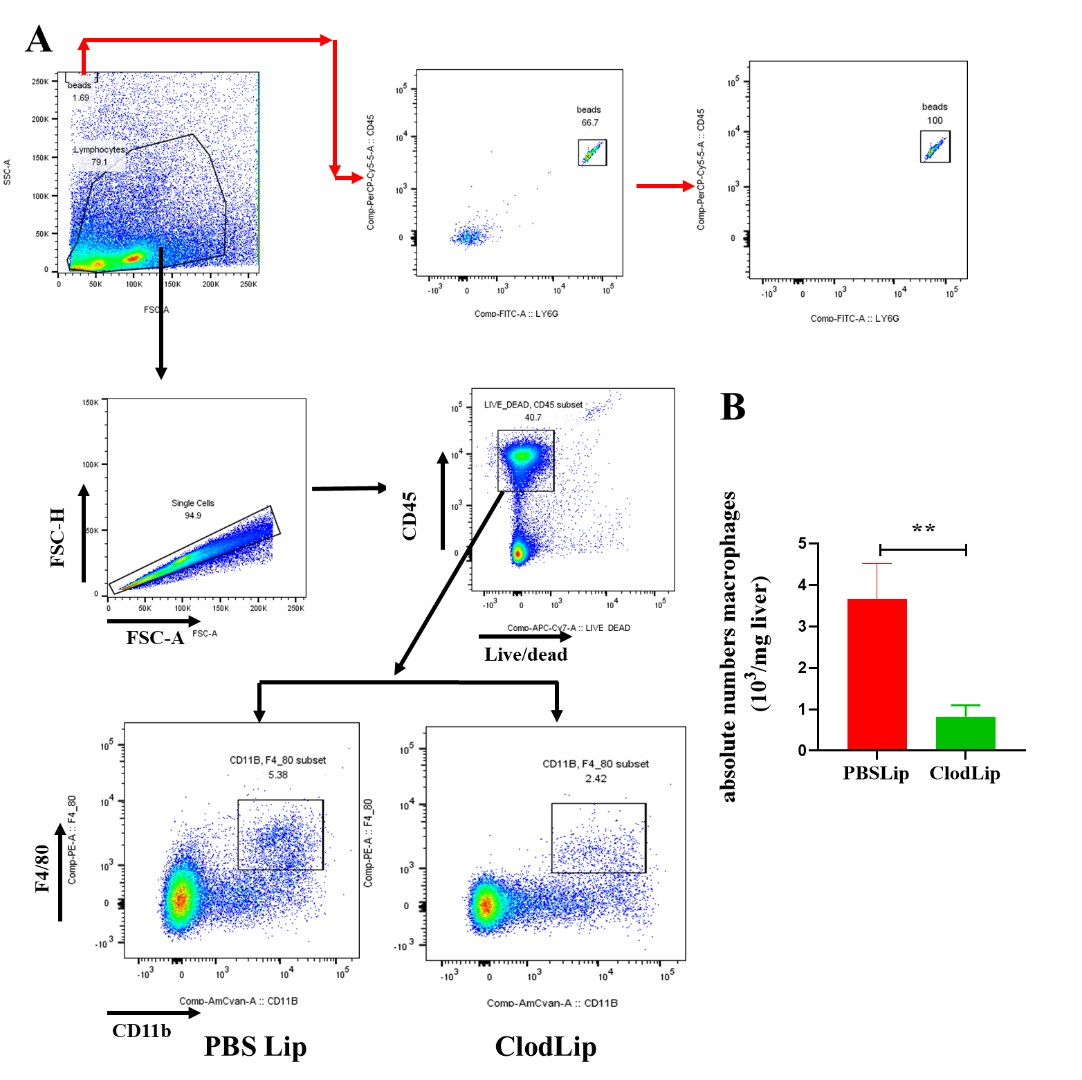


**S2 Fig. The depletion of macrophages in the liver of mice using clodronate liposome. (A)** the black arrows show the gating strategy of the macrophages. The red arrows show the signal of beads for counting cells. **(B)** the absolute numbers of macrophages in the liver after PBS liposomes (PBS Lip) or clodronate liposomes (ClodLip) treatment. N=4~6 mice per group. Compared with the corresponding group, ***P*<0.01, ns means no statistical differences.
